# Navigating the labyrinth of recurrent hematuria: a diagnostic puzzle

**DOI:** 10.11604/pamj.2026.53.58.49826

**Published:** 2026-02-04

**Authors:** Nirlipta Swain, Jayashree Bhawani

**Affiliations:** 1 Department of Pathology, Jawaharlal Nehru Medical College, Datta Meghe Institute of Higher Education and Research, Sawangi (Meghe), Wardha, Maharashtra, India

**Keywords:** Transitional, bladder, hematuria, lipoblast, smoking

## Image in medicine

Urothelial carcinomas, commonly termed transitional cell carcinomas, constitute one of the most common malignancies of the urinary tract. Bladder cancer ranks as the ninth most common type of cancer globally, comprising 3.1% of all cancers. Invasive urothelial carcinoma constitutes roughly 90% of primary bladder tumours with a male preponderance. They develop from the urothelium, the specialised epithelial lining of the bladder, ureters, renal pelvis and urethra. They are termed ‘transitional’ because of their distinctive ability to stretch and change shape. Etiological factors include exposure to urinary carcinogens, especially those derived from smoking. Hematuria is frequently encountered in clinical settings in these patients. The lipid-rich variant of urothelial carcinoma is an exceedingly uncommon and rapidly growing subtype. Fewer than 40 cases have been recognised currently. Microscopically distinguished by large lipoblast-like cells containing clear cytoplasmic vacuoles, which indent the nuclei, consisting of 10-50% of the tumour. We describe the case of an 82-year-old hypertensive male patient presenting with intermittent hematuria, urinary frequency, urgency and nocturia. Computed tomography (CT) urography demonstrated a neoplastic mass lesion along the anterior bladder wall. Transurethral resection of bladder tumour (TURBT) was performed, and tissue was sent for histopathology. Microscopy revealed pleomorphic lipid-containing vacuolated cells imparting a lipoblast-like appearance encroaching into the muscularis propria; these features are consistent with invasive lipid-rich urothelial carcinoma. The patient received adjuvant chemotherapy and radiotherapy and recovered smoothly. Attributing to its infrequency, aggressiveness, and poor outlook, early detection is paramount for management and close follow-up.

**Figure 1:**
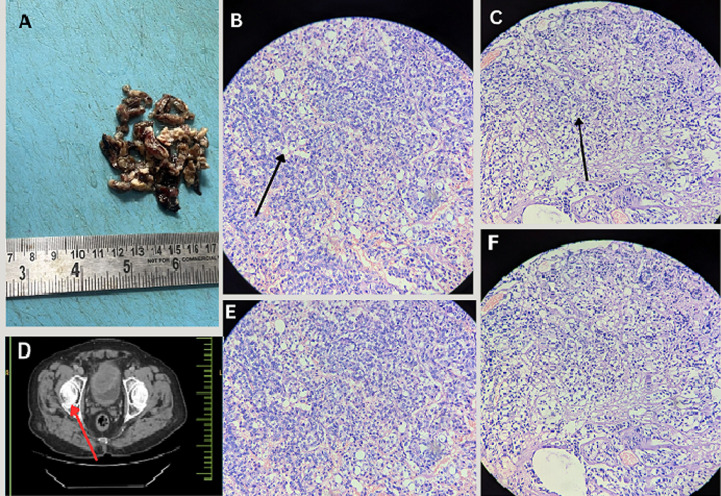
A) gross picture of the excised specimen (TURBT); B) microscopy demonstrating lipoblasts-like cells with one or more cytoplasmic vacuoles indenting the nuclei (black arrow) 40x H&E; C) microscopy showed neoplastic cells with eccentrically placed nuclei (black arrow) 20x H&E; D) CT urography revealed a neoplastic mass in the anterior wall of the urinary bladder (red arrow) along with grade 1 prostatomegaly; E, F) microscopy demonstrating cells exhibiting intermediate nuclear grade with pleomorphism 40x H&E and 20x H&E

